# Exercise addiction: A narrative overview of research issues

**DOI:** 10.1080/19585969.2023.2164841

**Published:** 2023-01-20

**Authors:** Aviv Weinstein, Attila Szabo

**Affiliations:** a The Isadore and Ruth Kastin Chair for Brain Research, Department of Psychology, University of Ariel, Ariel, Israel; b Institute of Psychology, and Institute of Health Promotion and Sport Sciences, ELTE Eötvös Loránd University, Budapest, Hungary

**Keywords:** Behavioural addiction, compulsive exercise, depression, eating disorders, exercise dependence

## Abstract

This narrative overview summarises the work on exercise addiction (EA) over the past 12 years and exposes critical conceptual and methodological issues. More than 1000 articles exist on EA, conceptualised as uncontrolled training harming the individual. Still, EA has no clinical diagnosis criteria at this time. Research is increasing continuously, but it is stale in advancing knowledge. Scalar measurement and lack of differentiation between *addictive* and *instrumental* exercise could be reasons for insufficient progress. Exercise addiction fits in the framework of behavioural addictions, but excessive exercise patterns also co-occur with other morbidities, including eating or body-image disorders. In these cases, exercise is instrumental; it functions to achieve a non-exercise-related goal. Therefore, it is essential to separate primary from secondary EA. Based on the interactional model, significant stress and capacity-exceeding ambitions fuel primary EA, while chief motives behind secondary EA embed body image dissatisfaction and eating disorders. Few reports exist on EA’s brain mechanisms, which could delay its classification as a distinct psychiatric dysfunction. Treatment of EA involves cognitive-behavioural approaches, but we know little about their effectiveness. Conceptually focussed psychophysiological research and in-depth interviews, complementing scalar data, could answer several open questions in this widely studied but relatively stagnant scholastic field.

## Introduction

### Scope of this review

**Figure 1.**
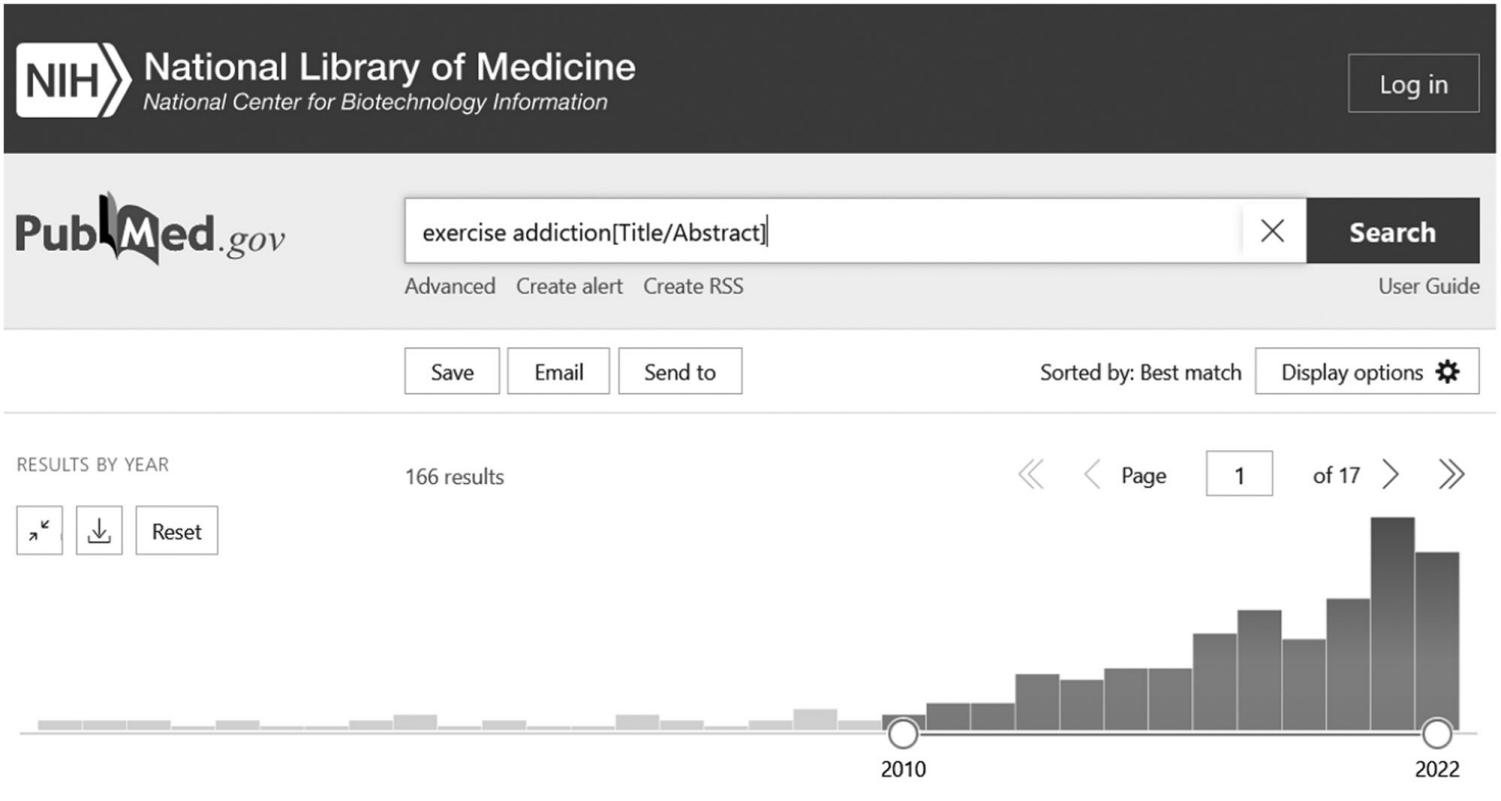
Exercise addiction abstracts PUBMED

Exercise addiction (EA) is a dysfunctional behaviour characterised by exaggerated training, loss of control over exercise behaviour, and negative life consequences that could be physical, psychological or social, or a combination of the three (Juwono and Szabo 2021; Szabo and Demetrovics 2022). Research interest in EA is continuously growing (Figure 1). Most growth occurred within the past 12 years, culminating in the last two years (Figure 1). However, despite its known dysfunctional characteristics, EA is still not recognised as a distinct category of psychiatric morbidity in the clinical reference manual, the Diagnostic and Statistical Manual of Mental Disorders (DSM-5; American Psychiatric Association 2013). The lack of inclusion is due to insufficient evidence for consistent aetiological and symptomatic manifestations (Szabo and Demetrovics 2022). Indeed, the various correlates of EA in different cases (Juwono and Szabo 2021) made Szabo (2010) believe that EA is a symptom of other psychiatric dysfunctions. In this focussed thematic narrative overview, we intend to highlight the factors hindering progress in this research area.

Figure 1 shows the growth of publications over the past 12 years on exercise addiction based on title and abstract search of a medical database (screenshot from PubMed).

### Definition

While sports, exercise, or regular physical activity are physically and psychologically beneficial, sometimes overindulgence in sports or physical activity could be harmful. We talk about dysfunctional practice when one exhibits an exaggerated training volume and loses control over the exercise behaviour, eventually harming oneself. Many psychological and physiological theoretical models exist for dysfunctional forms of exercise (Szabo and Demetrovics 2022). Like other known addictions, exercise addiction (EA) has six components: salience, tolerance, mood modification, withdrawal, conflict, and relapse (Griffiths 2005). Furthermore, EA involves the obsessive-compulsive aspects (Szabo and Demetrovics 2022). Therefore, it lies within the obsessive-compulsive and impulsive spectrum of behavioural addictions (Szabo and Demetrovics 2022). Additionally, EA involves physical symptoms such as withdrawal and is associated with social, medical, and financial problems (Berczik et al. 2012; Weinstein and Weinstein 2014).

Finally, EA earlier was classified as either primary or secondary (de Coverley Veale 1987). A primary EA develops in the absence of other disorders. It involves dependence and compulsive exercise, and the reward is *directly* associated with fulfilling the activity. On the other hand, secondary EA surfaces in another established morbidity, such as eating disorders, including anorexia nervosa and bulimia nervosa, or various body-image dysfunctions (Zou et al. 2022). In secondary EA, the high exercise volume is *instrumental* in achieving a non-exercise-related objective; hence, the reward is only *indirectly* associated with exercise fulfilment. Therefore, Szabo and Demetrovics (2022) have suggested referring to secondary EA as an ‘instrumental’ exercise form to unlink it from addiction and, thus, to avoid a significant conceptual confound in the field of study.

***Identified problem:*** Addiction and overexercising for other reasons are often confounded.
***Solution*:** Assess EA and use other measures/interviews to identify instrumental exercise.

### Method of data extraction

This narrative overview synthesises information from articles on EA published between January 2010 and September 2022, the period containing most research publications in the field. Keywords were entered in PubMed and Scopus databases using ‘exercise addiction’ and ‘sports addiction’. The database searches yielded 681 records. Located articles were screened for suitability using inclusion criteria focussing on the intended subtopics forming the infrastructure of the current review. They included: (1) assessment of EA, (2) prevalence rates, (3) physiological characteristics, (4) comorbidities, (5) emotional factors, (6) passion, (7) cognitive factors, (8) gender, and finally, (9) treatment. These subtopics form the structure of this overview, which is based only on English publications in peer-reviewed journals focussing specifically on EA. It excludes abstracts, dissertations, methodological papers, and conference papers. Based on the titles and abstracts of the located articles, we excluded 535 records and reviewed 146 articles using these criteria. However, we only use 69 studies and reviews in this overview due to DCNS’ limitation in reference numbers.

### Assessment of exercise addiction

First, we emphasise that currently, there is no clinical diagnosis for EA, as discussed above. Instead, scales or questionnaires measure dysfunctional attitudes and/or practices in one’s training on a spectrum ranging from low (asymptomatic) to high (symptomatic). The higher scores on these instruments can be associated with either primary or secondary EA. Because research seldom distinguishes between the two, secondary EA, in which exercise is instrumental in fulfilling a need unrelated to exercise, is also treated within the addiction framework. Consequently, the incidence of EA is likely to be overestimated.

Several questionnaires exist for assessing EA. They emerged over the past 30 years (Berczik et al. 2012). A review of 17 instruments suggests a strong dichotomy concerning the primary or secondary character of the problematic exercise (i.e., either the origin of the problem is within the behaviour itself or, conversely, within another disorder). This dichotomy limits the extant tool’s capacity to adequately capture this construct’s multidimensionality (Trott et al. 2020; Sicilia et al. 2021). Trott et al. (2020) have suggested that studies on EA should simultaneously measure eating disorders because false results may emerge without such measures.

Szabo and Demetrovics (2022) have warned that these instruments are not diagnostic tools because even those who report high scores may not exhibit dysfunctional exercise. Therefore, results based on these measures may contribute to the overpathologizing of EA. Indeed, when questionnaires are validated with interviews, the former overestimates the incidence of EA (Müller et al. 2014). Therefore, this widely or almost exclusively used EA assessment method, aimed at inference to pathology, has several shortcomings (Alcaraz-Ibáñez, Paterna, Sicilia, et al. 2022). In their book, Szabo and Demetrovics (2022) have evaluated the most extant instruments. They conjectured that the high inconsistency in the measured prevalence of EA stems from the different samples studied with various tools having different focuses, conceptualizations, and theoretical underpinnings. Hence, they propose in-depth interviews for spotting EA.

***Identified problem:*** Scalar data yield no diagnosis, and their variety yields mixed results.
***Solution:*** Use fewer but more reliable assessment tools along with interview follow-ups.

### Prevalence rates

The prevalence estimates stem from questionnaire data, which often may only reflect high commitment, dedication, or passion associated with one’s exercise (Szabo and Demetrovics 2022). Therefore, their interpretation is questionable because their confounding results could falsely project a higher than the actual occurrence of EA. However, handicapping cases of exercise addiction exist based on the few published case studies in academic works (refer to Szabo and Demetrovics (2022)) and testimonials collected on the internet (Juwono and Szabo 2021). Nevertheless, the number of established cases of EA is meagre and may only represent a fraction of the reported prevalence estimates.

Four academic survey years between 2016 and 2020 with 8251 participants of the national (USA) Healthy Minds Study (Ganson et al. 2022) showed that among participants, 11% of men and 17% of women reported compulsive exercise. (Given that compulsion is one constituent of addictions, dependence being the other (Szabo and Demetrovics 2022), neither compulsive nor dependent exercise can be defined as EA.) Compulsive exercise was observed in men and women college students and was associated with substance use behaviours and poor mental health symptoms (Ganson et al. 2022). A meta-analysis of 13 studies on EA, including 3635 participants, revealed an overall prevalence rate of 6.2%. The rate among amateur competitive athletes was 5.0%, 5.5% among university students, and 8.1% among general exercisers (Trott et al. 2020). A further meta-analysis on the prevalence of EA with and without eating disorders (nine studies with 2140 adult participants) has revealed that EA occurred more than three and a half times as often as a comorbidity to an eating disorder than in people without eating disorders (Trott et al. 2021). Therefore, this meta-analysis suggests that around three-thirds of the studies reporting EA prevalences may only measure instrumental exercise or secondary EA.

Concerning specific exercise activities, a study on the EA (see Table 1) found that 5% of individuals practicing cross-fit are at high risk (Lichtenstein and Jensen 2016). This figure was 13.3% among indoor cyclists (Bueno-Antequera et al. 2020), 4.4% among Italian runners (Zandonai et al. 2021), 15.4% among marathon runners (Collado-Boira et al. 2021), 7.6% among elite athletes from 15 sports disciplines competing at a national level (Lichtenstein et al. 2021), 6.1% among amateur endurance cyclists (Bueno-Antequera et al. 2022), and 7% among marathon runners (Lassner et al. 2022). In addition, a literature review up to 2018 has revealed that the highest EA occurs among endurance athletes (14.2%), followed by ball game players (10.4%), fitness centre attendees (8.2%), and power disciplines (6.4%), compared with 3.0% in the general public (Di Lodovico et al. 2019). There are also national differences. For example, EA is between 3% and 3.6% in Britain, 8.5% among Italian adolescents, and 0.3 to 0.5% of the general adult population in Hungary (Berczik et al. 2012). In the exercising population, the EA appears to be around 3.0% (Sussman et al. 2011), but as Szabo and Demetrovics (Szabo and Demetrovics 2022) warn, these figures do not reflect pathology. In contrast, they may mirror strong dedication, high commitment, and passion for the adopted sport or exercise activity. See Table 1 for the prevalence of exercise addiction among athletes.

**Table 1.** Prevalence of exercise addiction among athletes (arranged chronologically).

Study (year and reference number)	Country and study population	Number	Prevalence rates	EA definition and assessment
Lichtenstein and Jensen (2016)	Denmark	603 Cross-fit	5%	EAI – An online survey using Facebook groups.
Bueno-Antequera et al. (2020)	Spain	1014 Indoor cyclists	13.3%, high risk	EAI using a web-based experiment.
Zandonai et al. (2021)	Italy and Japan	229 Italian and 198 Japanese runners	4.4% among Italian runners and 0% among Japanese runners	EDS-R using an interview.
Collado-Boira et al. (2021)	Italy	317 Runners, 31.9% road marathon, 28.7% mountain marathon, 39.4% ultra-trail	15.4%	EDS-R and observation.
Lichtenstein et al. (2021)	Denmark	417 Elite athletes from 15 sports disciplines	7.6%	EAI using an online survey.
Ganson et al. (2022)	USA	8251 Participants of the national (USA) Healthy Minds Study	11% of men and 17% of women	Compulsive exercise was measured based on number of occurrences in the past 28 days (analyzed continuously and among those who reported ≥1 and ≥20 occurrences). Using a national survey.
Bueno-Antequera et al. (2022)	Spain	330 Adult cyclists (30 women)	6.1%	EAI using a survey from pre- to 6-month post-competition.
Lassner et al. (2022)	Germany	72 Marathon runners (19.4% female)	6.94%	EAI, using a survey with physiological measures.

Abbreviations and references: EAI: Griffiths et al. (2005); EDS-R: Downs et al. (2009).

Because these prevalence rates, in most cases, include primary and secondary EA in various samples, they have little validity (Trott et al. 2020). Therefore, unselective assessment raises a crucial issue in the interpretation of many studies because, in primary EA, reasons beyond and associated symptoms (or their severity) of the dysfunction could be different than in secondary EA, in which exercise assumes a secondary role. Based on the interactional model by Egorov and Szabo (2013), primary EA occurs when exercise is the means of coping with stress or trauma and reflects an escape from hardship. Alternately, it could stem from overestimating one’s limits on the path of athletic achievement. However, questionnaire data-based results inflate estimates *via* the scores of those who use exercise to achieve another goal, like losing weight or changing body shape. Consequently, future research should study EA without and with comorbidities separately to avoid considering instrumental exercise as an addictive behavior (Trott et al. 2020).

***Identified problem:*** Due to conceptual and assessment issues, prevalence rates are inexact
***Solution:*** The prevalence rates should be based on clearly identified dysfunctional cases.

### Physiological characteristics of exercise addiction

Only a few studies looked at the physiological correlates of EA. Sporadic research suggests resting leptin levels (adjusted for body fat) correlate with primary EA symptoms (Lichtenstein et al. 2015). Another work examined 50 professional football players during seasonal training and a 7-day exercise deprivation. The results showed that during the deprivation period, athletes with high EA had lower brain bioelectric activity (lower α-rhythm amplitude and power), increased muscular tension, augmented sympathetic activity, and increased anxiety and depression (Krivoschekov and Lushnikov 2011). Furthermore, in a study of 53 male athletes, EA correlated with eating disorder symptoms, negative energy balance, and higher cortisol levels (Torstveit et al. 2019). Finally, a survey of 176 people who performed aerobic and anaerobic exercises at least three times a week found no difference between aerobic and non-aerobic exercisers in the EA or additional symptoms (Pálfi et al. 2021). However, these findings are only sporadic and unrelated. Therefore, their results cannot be generalised to identify the physiological correlates of EA. Indeed, there is a shortage of studies examining EA's psychophysiological and neuroendocrinological aspects. This void is a limitation in understanding this dysfunction’s clinical nature.

***Identified problem:*** There is a lack of focussed research on the physiological aspect of EA.
***Solution:*** More systematic and focussed research is needed in the area.

### Comorbidity with other psychiatric disorders

#### Eating disorders

Exercise addiction has high comorbidity with eating- and body-image disorders (Sussman et al. 2011). To assess this connection, a meta-analysis of 36 studies with 21,816 participants has found a small-sized relationship between symptoms of bulimia, a small to medium-sized relationship between body and eating concerns, and a medium relationship between overall eating disorder symptoms and dietary restraint. In particular, clinical, younger, and thinner samples showed a higher prevalence of eating disorder symptoms (American Psychiatric Association, 1994; Alcaraz-Ibáñez et al. 2020). Table 2 shows the comorbidity studies of EA. These comorbidities often include instrumental exercise and may not mirror addictive exercise behaviour.

**Table 2.** Co-occurrence of exercise addiction and psychiatric symptoms and disorders (arranged chronologically).

Study (year and reference number)	Country	Number	Co-occurring disorders/symptoms and rates or association with psychopathology	Assessment method
Weinstein et al. (2015)	Israel	20 Professional regular exercisers	Anxiety, depression	CES, BDI, STAI
		51 Recreational regular exercisers		
Li et al. (2015)	China	1601 College students	State anxiety, depression	EAI, STAI, CES-D, SWB.
Levit et al. (2018)	Israel	100 Competitive and amateur athletes (67 males and 35 females)	Depression and abnormal eating attitudes	EAI, EAT, STAI, BDI.
Lichtenstein et al. (2018)	Denmark	1083 Recreational exercisers	Depression, stress	EAI youth version
				SCOFF
				EA prevalence of 21% in patients with eating disorders.
Pinto et al. (2019)	Israel	288 Combat reserves, non-combat reserves and control participants	Stress	EDS-R, EAI-Revised, PSS
Rogers et al. (2019)	USA	540 Participants from the general public	Depression, suicide attempts	EDS, ACSS
				History of suicidal behaviour.
Meyer et al. (2021)	Switzerland	156 Individuals who exercise more than 10 h a week	Depressive disorders, personality disorders and OCD	EDS, SCID-5-CV, SCID-5-PD. An interview based on the DSM-5 criteria of non-substance-related addictive disorders to explore the severity of exercise addiction symptoms.
Bueno-Antequera et al. (2020)	Spain	330 Cyclists	Depression and anxiety	IPAQ short form, EDI and health outcomes through a web-based experiment.
Lassner et al. (2022)	Germany	72 Marathon runners (20% female)	Depression	EAI, GAF, IPAQ, BDI, PANAS
Gunnarsson et al. (2022)	Sweden	3029 responders to fitness media	Social phobia, eating disorders and OCD	EAI, AUDIT-C, Yes/no questions regarding a variety of psychiatric disorders
Alcaraz-Ibáñez, Paterna, Griffiths, et al. (2022)	Spain	691 Undergraduate leisure exercisers	Depression	EAI, the Depression sub-scale of the BSI-18, SCOFF
Colledge et al. (2022)	Switzerland	123 Individuals exercising for more than 10 h a week	Depression, ADHD and childhood trauma	EDS, BDI, HASE, ADHS, CTQ
Habelt et al. (2022)	Austria and Germany	335 Participants, 88 with mountaineering addiction and 247 control participants	Depression, anxiety, eating disorders, alcohol abuse or dependence, illicit drug abuse	EAI, EDS adapted version for mountaineering, PHQ-4, PHQ-9, body mass index BMI.

Abbreviations and references: ACSS: Bender et al. (2011); ADHS: Schmidt et al. (2018); AUDIT-C: Bush et al. (1998); BDI: Beck et al. (1996); BSI-18: Derogatis (2000); CES: Tuttle (1992); CES-D: Lewinsohn et al. (1997); CTQ: Bernstein et al. (1994); EAI: Griffiths et al. (2005); EDS: Hausenblas and Downs (2002); EDS-R: Downs et al. (2009); GAF: Start-up et al. (2002); EAT: Garner and Garfinkel (1979); IPAQ: Craig et al. (2003); PANAS: Watson et al. (1998); PHQ-4: Löwe et al. (2010); PHQ-9: Levis et al. (2019); PSS: Cohen et al. (1983); SCID-5-CV: First et al. (2016); SCID-5-PD: First et al. (2015); SCOFF: Morgan et al. (1999); STAI: Spielberger et al. (1983); SWB: Ji and Li (2006).

***Identified problem:*** Excessive exercise is an EA confounding symptom in eating disorders.
***Solution:*** Screening for and excluding eating-disordered cases in studying EA.

#### Anxiety, depression, and obsessive compulsive disorder (OCD)

Exercise-addicted individuals who exhibit obsession or compulsion with physical activity are often present among individuals who exercise longer than 10 h a week. They show a high prevalence of depressive disorders (56%), personality disorders (47%), and obsessive-compulsive disorders (31%). Meyer et al. (Meyer et al. 2021) have argued that EA is more characterised by obsessive-compulsive rather than impulsive traits, which is in accord with Szabo’s and Demetrovics’s (Szabo and Demetrovics 2022) recent classification on the compulsive-impulsive spectrum.

Several studies have reported comorbidity with anxiety, depression, and abnormal eating attitudes (Weinstein et al. 2015; Levit et al. 2018). For example, in a sample of Chinese college students, EA was associated with state anxiety, depression, and subjective well-being (Li et al. 2015). Furthermore, in another study, combatant Israeli army reserves reported higher intensity and longer periods of exercise while having higher EA scores than reserves in non-combatant roles and control participants (Pinto et al. 2019). Finally, in another work, EA was strongly associated with eating disorders, compulsiveness, and anxiety disorders (Gunnarsson et al. 2022).

Among recreationally exercising undergraduate students, depressive symptoms have explained a significant portion of the variance in EA symptoms. On the other hand, EA symptoms have accounted for substantial variance in depression symptoms. It seems that symptoms of depression and EA have a bi-directional relationship, at least in leisure exercisers (Alcaraz-Ibáñez et al. 2022). Recreational exercisers with increased EA had more depression and stress symptoms, which worsened after injury (Lichtenstein et al. 2018). Those who have attempted suicide continued to exercise despite physical or psychological harm and lack of control, at the expense of other activities, compared with those who did not try suicide (Rogers et al. 2019).

There is evidence for higher anxiety symptom severity among women cyclists with high EA scores (Bueno-Antequera et al. 2020). It is also related to depression, ADHD, and childhood trauma (Colledge et al. 2022). Also, individuals who practice extreme mountaineering show higher ratings of self-perceived stress, depression, anxiety, eating disorders, alcohol abuse or dependence, illicit drug abuse, and current and history of psychiatric disorders (Habelt et al. 2022). Among marathon runners, higher values of EA correlated with a reduced level of general functioning, depressive symptoms, and negative affect (Lassner et al. 2022). Again, these studies do not segregate addiction to exercise *per se* from what Szabo and Demetrovics (Szabo and Demetrovics 2022) call *instrumental exercise*. These authors argue that secondary EA is not an addictive behaviour; therefore, *instrumental exercise* (p. 127) is a more appropriate term for describing the phenomenon.

***Identified problem:*** The *direction* of EA’s relationship with comorbidities is unclear.
***Solution:*** Longitudinal and epidemiological studies are needed to clarify this issue.

#### Body image, appearance, and self-esteem

Several studies have investigated the risk and protective factors of EA. Among 288 regular exercisers, there were positive correlations between Alexithymia and EA, mediated by body image concerns. Furthermore, self-esteem showed a relevant moderating effect, such that at high levels of self-esteem, the impact of Alexithymia on body image concerns became insignificant (Gori et al. 2021). In 1689 Chinese college students with an extended exercise history, appearance- and ability-motivation were directly associated with EA, and competence need was directly related to exercise dependence (Li 2018). Body dissatisfaction appears to be highly related to EA (Alcaraz-Ibáñez et al. 2021). In these studies, exercise might be instrumental in achieving a related goal.

Further, an association emerged between the time spent on vigorous physical activities and exercise levels during the second wave of the Covid-19 pandemic, and body image was positively related to EA (Bonfanti et al. 2022). However, considering the interactional model by Egorov and Szabo (2013), the fear of getting sick from Covid-19 and radical changes in lifestyles induced by the lockdown and individual personal characteristics may have helped the development of EA (Ceci et al. 2022). Acting as a coping mechanism, the fulfilment of exercise yielded relief and, thus, became obligatory because a lack of training would cause negative results. In this case, the behaviour fits the interactional model and reflects EA as a coping means.

***Identified problem:*** Overexercising in body-image disorders is instrumental, not addictive.
***Solution:*** The *main reason* for exercise needs to be determined in body-image disorders.

### Personality

Several studies have found an association between narcissism, perfectionism, and EA (Birche et al. 2017). For example, among 90 regular exercisers recruited from various gyms, fitness centres, and sporting events, the EA was positively related to narcissism, self-orientated perfectionism, and socially prescribed perfectionism (Miller and Mesagno 2014). In a study of 241 amateur endurance athletes, men had high ratings of narcissism (grandiosity feelings) and psychopathy (coldness) factors. Higher traits of narcissism and Machiavellianism increased the likelihood of EA (Nogueira et al. 2019). All features of narcissistic personality correlated positively with EA in an Israeli community sample (Zeigler-Hill et al. 2021). In a recent review, perfectionism was weakly or moderately related to EA (Çakın et al. 2021). Finally, another review reported that both perfectionism and narcissism are related to EA (González-Hernández et al. 2022).

A study of 642 participants in the UK has shown that problematic exercise is related to impulsive and compulsive personality features, emotional dysregulation, and disordered eating (Chamberlain and Grant 2020). In another research conducted with 140 endurance athletes, exercise dependence was inversely related to personal resources trait, state self-control, and self-concordance but not social support (Zimanyi et al. 2021). A further study reported that Alexithymia and two forms of impulsivity (rash impulsiveness and reward sensitivity) were predictors of EA. However, mediation analysis showed that Alexithymia was the strongest predictor and fully mediated the contribution of rash impulsiveness (Lyvers et al. 2021). In a recent study, EA positively correlated with reward sensitivity and interoceptive awareness (Lyvers et al. 2022), and participants with severe bingeing scored higher on EA, Alexithymia, risky alcohol use, and sensitivity to reward and punishment. Furthermore, extraversion and narcissism, but not agreeableness, appear to be associated with EA (Cook et al. 2020). Finally, negative urgency and sensation seeking were also related to EA (Kotbagi et al. 2017). Therefore, certain personality traits could be associated with EA and dysfunctional exercise. Still, it is unclear how and under what conditions this relationship has a weaker or more substantial prevalence and impact. Consequently, more controlled research is needed to understand personality’s role in exercise addiction.

***Identified problem:*** Personality appears to be related to EA, but few studies support it.
***Solution:*** More focussed theory-driven research on personality and EA is needed.

### Emotional factors

Emotional factors could be said to play a role in all types of psychological dysfunctions. Indeed, emotions of shame, guilt, and pride correlated with EA in 296 runners (Sicilia et al. 2020). In addition, compulsive exercise was associated with women’s exercise volume and emotional components (Ruiz‐Turrero et al. 2022). Further, individuals having high EA scores and muscle dysmorphia showed ineffective decision-making strategies and selected inappropriate goals for physical exercise with negative consequences (Olave et al. 2021). These studies are only a fraction of the research studying various connections between emotional components and dysfunctional training. A primary emotion strongly connected to EA is the combination of stress-related emotions. Stress is part of many models accounting for the aetiology of exercise addiction (Szabo and Demetrovics 2022). However, research on EA is somewhat sporadic, and only a few studies have been replicated. More systematic investigations and case-study analyses are needed, with a focussed classification of the emotions and their connection to EA, with the exclusion of instrumental exercise.

***Identified problem:*** How and when EA is affected by various emotions is unclear.
***Solution:*** Qualitative and case studies are needed to understand the quantitative work.

### Obsessive and harmonious passion

Recently, studies have evaluated obsessive and harmonious passion as predictors of EA. Exercise intensity, obsessive passion, and harmonious passion significantly predicted the EA (with obsessive passion being the most robust predictor) among 360 individuals with a long exercise history. Furthermore, women exhibited more obsessive and harmonious passion than men with similar exercise intensities (Kovacsik et al. 2019). Obsessive and harmonious passion were significant predictors of EA in 190 teams-versus individual exercisers (Kovacsik et al. 2020). Finally, a latent growth modelling of 149 women and men who had just started a new sport activity showed that the EA and passion were high at baseline and increased over the 12 weeks. The EA was predicted by being female, team sport participation, higher exercise intensity, and identified motivation (Kovácsik et al. 2021). Similar longitudinal studies are needed to understand EA better. Szabo et al. (2022) have argued that harmonious and obsessive passion are independent predictors of EA, and when they are controlled, EA-related group differences often vanish.

***Identified problem:*** Passion for exercise may predict or confound EA measures.
***Solution:*** Studies should test the extent to which EA assessment is affected by passion.

### Cognitive function, event-related potentials (ERPs), and brain imaging studies

There are few studies on the cognitive and brain mechanisms associated with EA. A study that investigated frontal executive functions with ERPs in exercise-addicted, moderate exercisers, and exercise avoiders found that ERP components P3 and N2 latencies were shorter in the EA group than in the other groups. Furthermore, exercise-addicted and moderate exercisers showed faster response time than exercise avoiders (Ryu et al. 2016). Another research on badminton players with high and low scores of EA, performing the oddball paradigm using Go/NoGo tasks with auditory stimulation and Event-related potentials, showed that the low EA group exhibited a larger P300 amplitude than the high EA group. In contrast, P300 latency was shorter in the high EA group than in the low EA group (Kwon et al. 2018). In addition, the N200 amplitude was smaller in the low EA group than in the high EA group, while N200 latency was shorter in high EA groups than in low EA groups (Kwon et al. 2018).

Additionally, a recent study investigated the effects of EA on the brain’s structure in 86 individuals who exercise regularly by estimating their cortical gray matter volume (GMV) in structural magnetic resonance imaging (MRI) (Zhang et al. 2021). Whole-brain analyses revealed a negative correlation between EA and GMV. This relationship emerged in three places: (1) right orbitofrontal cortex (OFC), (2) left subgenual cingulate gyrus (sgCG), and (3) left inferior parietal lobe (IPL). Furthermore, the GMV of the right OFC was a mediator between stress and EA (Zhang et al. 2021). These few studies suggest that some correlates exist between neuropsychophysiological measures and exercise addiction, but significantly more focussed research is needed in this field of study.

***Identified problem:*** Neurophysiological studies are promising in the study of EA.
***Solution:*** More neuro-/psychophysiological studies are needed in this area of research.

### Gender differences

Gender differences in measures of dysfunctional symptoms, quality of life, mood, and sleep were reported among athletes. Males showed higher dedication to training and vigour compared with females. Both genders had depression symptoms, although ratings were more elevated in females (Modolo et al. 2011). It was suggested that in women, there is a continuum of women’s exercise experiences, ranging from routine to more extreme regimens (Johnston et al. 2011). The need for body control increased excessive exercise and disturbed eating, whereas training was preferred over eating problems (Johnston et al. 2011). A review of 27 studies on EA and sex differences has found an effect size (Cohen’s *d*) between .04 and .98, suggesting that men are more addicted to exercise than women (Dumitru et al. 2018). However, a recent study indicated that when a minimal weekly volume of training (i.e., three (3) hours per week) is set as a criterion for participant recruitment, no gender differences are evident in EA (Szabo et al. 2022).

***Identified problem:*** Gender differences are not examined under standardised conditions.
***Solution:*** Comparison of gender differences in EA requires standardised protocols.

### Treatment

The primary treatment for dysfunctional exercise behaviour is based on the cognitive-behavioural approach, but there is currently little evidence of its effectiveness. Assessment and treatment for exercise addiction that requires clinical intervention should assess the stages of addiction and motivation to change, comorbidity with other psychiatric disorders, mainly eating disorders or substance abuse and alcohol abuse disorders (Freimuth et al. 2011). An evaluation of treatment strategies for high-performance athletes, including mindfulness-based interventions and cognitive-behavioural therapies (exposure and response prevention, cognitive restructuring, and behavioural experiments), has led to the argument that there is little evidence of the efficacy of treatment for EA (Martenstyn et al. 2021). A review has evaluated the use of rational emotive behaviour therapy (REBT) to treat EA (Outar et al. 2018). The results have indicated that treatment has reduced low-frustration tolerance, irrational beliefs, and EA scores and increased self-acceptance (Outar et al. 2018). Another review of the interventions for compulsive exercise in individuals with eating disorders (Hallward et al. 2022) reported improvements in both compulsive exercise and eating disorders in all 11 studies. The authors have argued that the findings support treating compulsive exercise as essential to treating eating disorders (Hallward et al. 2022). Finally, recently, Szabo and DemetrovicsSzabo and Demetrovics, 2022 have proposed a 10-step hierarchical treatment approach for people exhibiting dysfunctional exercise behaviour.

***Identified problem:*** Treatment is geared towards EA and comorbid problems like eating disorders.
***Solution:*** The idea that EA is a symptom of another mental disorder merits consideration.

## Discussion

Regular exercise positively affects physical and mental health, but overindulgent training may have adverse physiological and psychological consequences when practiced without control. There are controversial issues in defining, diagnosing, and establishing the aetiology of EA. When considering its classification, it is yet to be determined whether EA is a behavioural addiction or part of the OCD spectrum.

Although several valid and reliable questionnaires exist for measuring EA, these tools yield a scalar value that, at their higher ends, may or may not reflect dysfunctional exercise (Szabo and Demetrovics 2022). Further, studies on EA prevalence exhibit high variability. Their inconsistent findings are likely due to different assessment tools and heterogeneous samples. Furthermore, most studies report a higher prevalence of EA among men than women when there is no set criterion for exercise volume. Therefore, future research must compare genders at a standard exercise volume to obtain a more realistic picture of gender differences.

Several hypotheses explain the biological mechanisms of EA, including reward, endorphin, cognitive appraisal, sympathetic arousal, affect regulation, stress reduction, avoidance of withdrawal, obsessive-compulsive, and the interactional model (Egorov and Szabo 2013; Weinstein and Weinstein 2014; Szabo and Demetrovics 2022). There is also evidence from rodent studies that wheel running activates the dopamine reward system and can reduce stress. This mechanism may explain why physical exercise can improve positive moods and reduce negative emotions in humans (Weinstein and Weinstein 2014). Furthermore, running is also associated with endogenous endorphins and cannabinoids, resulting in ‘runners’ high’ or euphoria (Weinstein and Weinstein 2014). In general, EA is positively reinforced *via* positive affect, characterised by high arousal and positively altered feeling states. However, it is also fuelled by negative reinforcement when it aims to reduce stress and avoid withdrawal (Weinstein and Weinstein 2014). It is, therefore, argued that psycho-biological mechanisms can explain the positive and negative reward mechanisms typical of EA.

Several psychological correlates of EA have been exposed, including the intensity of the activity, body image or physical appearance, self-esteem, personality traits like narcissism, perfectionism, impulsivity, reward dependence, and emotional factors. In addition, situation-dependent factors like obsessive and harmonious passion have also been associated with EA. The degree to which EA overlaps with passion and the latter is predictive of dysfunctional exercise in various situations requires further research effort with a clear definition and valid assessment of EA. Despite the many correlates of EA, chronic stress is the foundation of many models of EA (Szabo and Demetrovics 2022).

Very few empirical studies exist on EA’s cognitive, pharmacological, and brain mechanisms. Indeed, little is known about the effects of excessive exercise on the brain’s structure and function. However, there is preliminary evidence of improved executive function, and its ERP correlates with changes in gray matter volume. Still, more studies are required to obtain a clearer image of the brain mechanism associated with EA.

There is increasing evidence for the comorbidity of EA with eating disorders, nicotine, and alcohol dependence. However, one study in a Norwegian sample found that EA is related to a lower prevalence of nicotine use (Szabo et al. 2018). There is also evidence of comorbidity with stress, anxiety and depression, body dysmorphic disorder, pathological buying, and hypersexual behaviour. The psychological treatment for EA relies on cognitive-behavioural approaches. Several studies have evaluated the effectiveness of cognitive-behaviour therapy and pharmacological treatment. However, their efficacy is still unclear because they may treat the various underlying reasons behind EA. A cognitive-behaviour therapy-based 10-stage hierarchical treatment model has been proposed recently (Szabo and Demetrovics 2022), but it needs to be tested in applied health settings.

### Limitations

There is high variability in prevalence rates of EA may be ascribed to the different questionnaires used. In addition, most studies are cross-sectional, and it is impossible to infer any causal effects of EA on physical and mental health. The field is in dire need of longitudinal studies. The current review’s limitation is that it is not a systematic review and only explores two databases. Therefore, this overview may not include all published articles on a specific subtopic. Still, we are confident that the general picture of the status of the research on EA is adequate, and adding other research reports would not change the conclusions. In our delimitations, we opted for a *thematic* narrative overview to illustrate why the advancement of knowledge is relatively stagnant despite increasing research output in the area. Therefore, we tried to identify and emphasise the most critical confounding issues likely to hinder research advancement in this area. We hope that we have accomplished our goal and that future empirical works will consider the research issues identified in this article.

## Conclusions

Exercise addiction has been researched for more than half a century. Still, the most intensive work in the area stems from the last half-decade. Most research has focussed on the definition, diagnosis, comorbidity, and characteristics of exercise addiction. Its assessment overwhelmingly fails to distinguish between addiction to exercise *per se* (primary EA) and dysfunctions in which excessive exercise is instrumental in achieving a non-exercise-related objective (also known as secondary EA). It is still not evident whether EA is a behavioural addiction or belongs to the obsessive-compulsive spectrum. Thus, it is still unclear whether EA is a distinct dysfunction or a common symptom of various underlying psychiatric morbidities. Due to the multiple different questionnaires used in assessing EA, its reported prevalence rates are likely inflated and overpathologize EA. The number of published case studies is low in academic literature, but significantly more cases may exist based on internet testimonials. Personality, gender, brain function, emotions, and several other correlates of EA need more systematic scrutiny to obtain a more transparent, stable, and easily replicable overview of the various relationships.

The very few studies on cognitive-behaviour and pharmacological treatment for EA do not enable us to estimate its effectiveness. Overall, research progress is stagnant mainly because of various tools of assessment, different theoretical models, and vastly different samples studied. A significant problem revolves around the differentiation between primary and secondary EA. Reviews suggest that the latter is more frequent than the former. Therefore, for the sake of conceptual clarification, there is a call for referring to secondary EA as ‘instrumental exercise’ because, in such cases, exercise is only a means to achieve a non-exercise-related objective. As such, it does not meet the criteria for classification as an addiction. Progress in the field depends on homogenising these issues and carrying out focussed research.
